# Achieving educational mission and vision with an educational scorecard

**DOI:** 10.1186/s12909-018-1354-4

**Published:** 2018-10-29

**Authors:** Jonathan Huntington, John F. Dick, Hilary F. Ryder

**Affiliations:** 1 Department of Medicine, Geisel School of Medicine at Dartmouth, Hanover, NH USA; 2 Department of Medicine and Medical Education, Geisel School of Medicine at Dartmouth, Hanover, NH USA; 3 The Dartmouth Institute for Health Policy and Clinical Practice, Geisel School of Medicine at Dartmouth, Hanover, NH USA

**Keywords:** Medical education, Faculty development, Performance measurement, Hospital medicine, Stakeholder engagement

## Abstract

**Background:**

Achieving an academic section’s educational mission and vision is difficult, particularly when individual faculty contributions may be self-directed and uncoordinated. Balanced scorecards have been used in other environments; however, a process for developing one focusing on the educational mission of an academic medical section has not previously been described. We aimed to develop and use an educational scorecard to help our academic clinical section achieve its educational mission and vision.

**Methods:**

Six medical educators participated in a task force that developed, implemented, and evaluated an educational scorecard that incorporates four domains of educational value and six stakeholder perspectives. A modified Delphi process using 14 experts built expert consensus on the most valuable metrics. The task force then developed performance targets for each metric.

**Results:**

Review of the scorecard at the sectional level resulted in both sectional and individual strategies which lead to a more balanced educational impact, including service structure changes and increased mentorship. Our section has used the scorecard and metrics to evaluate performance since 2014.

**Conclusion:**

An educational scorecard is a feasible way for academic groups to communicate educational goals, engage faculty, and provide objective information with which to base strategic decisions affecting their educational mission.

## Background

Academic departments often struggle to achieve their tripartite mission of research, clinical work, and education. Each faculty member’s contribution to this mission varies considerably. Demonstration of achievement is traditionally analyzed and rewarded at an individual level – through review of Relative Value Units (RVUs) generated, indirect costs awarded, publications submitted or teaching awards received. This uncoordinated focus at the individual level can prevent achievement of broader departmental or sectional goals as faculty, responding to varied incentives, are pulled in multiple directions. With increasing emphasis on clinical productivity, the educational mission receives less attention. As an academic section of Hospital Medicine, we sought to create a coordinated approach towards realizing our shared educational goals, seeking a method to create a shared vision, improve faculty engagement in education, and track our educational performance as a section.

In business, balanced scorecards are used as performance management tools to translate a mission and vision into specific objectives and metrics across multiple domains [1]. This allows for the monitoring of performance to ensure vision is achieved [2]. A scorecard is ‘balanced’ when it has a mixture of financial and non-financial perspectives, external and internal measures, leading (early) and lagging (late) performance indicators, and objective as well as subjective outcomes [3]. Balanced scorecards have been used in academic clinical departments [4], Graduate Medical Education [5], and teaching hospitals [6], but while frameworks to describe and track measures of clinical strength, financial health [7], research funding [8], quality improvement [9] or customer satisfaction have been described, similar methods to achieve the educational mission and vision are notably lacking. We describe our experience with the development and implementation of an educational scorecard focused on educational objectives that includes metrics that track performance towards the goal of achieving our section’s shared educational vision.

## Methods

### Setting and participants

Our Section of Hospital Medicine is embedded within a 396 bed academic tertiary care hospital with close affiliation with a small Ivy League medical school. Our 30 sectional faculty members share dual appointments at the hospital and medical school, and staff teaching services with internal medicine residents and third- and fourth-year medical students. We formed a task force of six faculty interested in refining the educational mission of the section, who varied in seniority, clinical full-time equivalent (FTE), and educational funding. We recruited fourteen experts representing different stakeholder perspectives, including patients, learners, educators, clinician-teachers and leaders at the medical school and residency level to assist us in the creation of our scorecard.

At the planning stage, our project was reviewed and approved by the Dartmouth College Institutional Review Board, the Committee for the Protection of Human Subjects on October 20, 2014 (STUDY00028408).

### Program description

As part of a strategic planning initiative, we developed and implemented an educational scorecard to assist in defining and achieving success in our educational vision and mission. As a necessary precursor to this process we developed and vetted formal educational mission and vision statements for our section using a process whereby a small group within the section created an initial draft which was subsequently edited and refined by increasingly larger numbers of faculty in an iterative process.

The group defined our educational mission as follows: “To decrease the burden of disease for our current and future patients through the design, implementation, facilitation and evaluation of educational activities. To foster curiosity, empathy and drive for personal improvement for medical learners of all stages. To create and foster a medical learning community to provide learners with knowledge, skills and attitudes to successfully overcome current and future challenges in health care.” Our educational vision was defined as follows: “To set the standard for excellence in medical education within the Department of Medicine and throughout the medical center. To be the learning community where all learn in an efficient manner and faculty can realistically build academically productive and meaningful careers.”

We were philosophically and theoretically inspired by the University of California-San Francisco Hospital Medicine Balanced Scorecard for Hospital Medicine [8] and by Ogrinc et al.’s [10] work describing the development of an Educational Value Compass. This conceptual framework had been developed to understand the value added to clinical care by educational activities in a balanced fashion across four distinct domains (Clinical, Functional, Satisfaction and Resources) from six different stakeholder perspectives from which the value of education should be assessed (Patients, Residency Programs, Clinician-Teachers, Learners, Medical Schools, and Community).

### Scorecard development

**Fig. 1:**
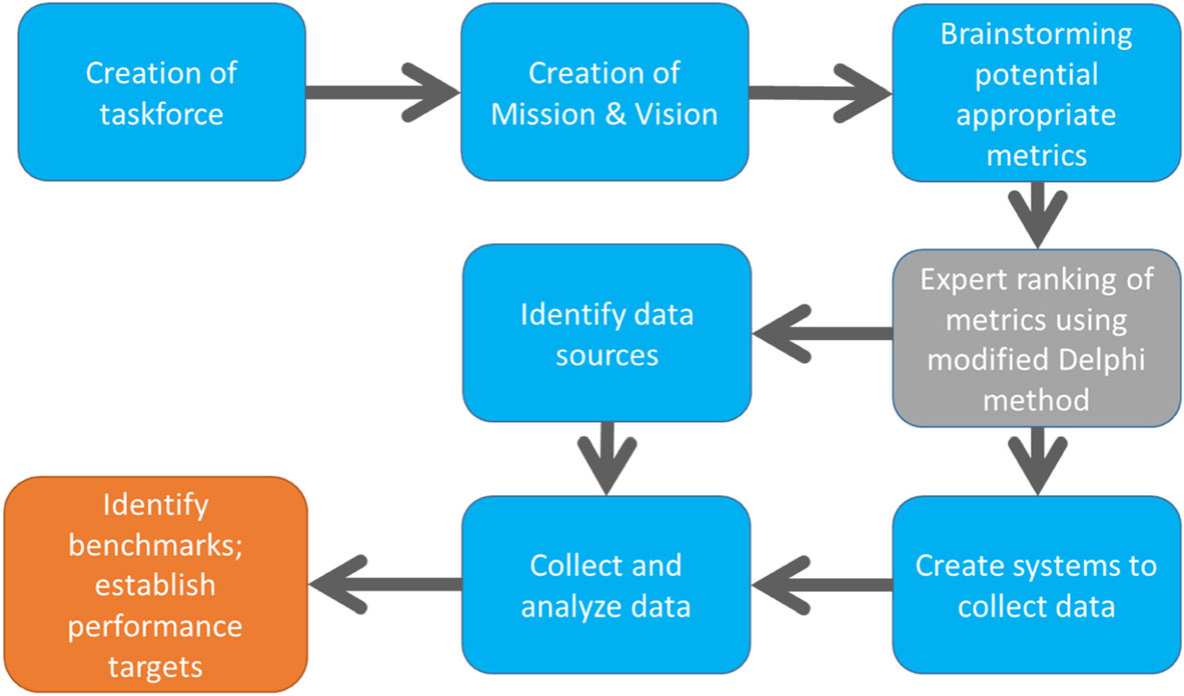
Educational Scorecard Development Process: High-level process map diagraming the process we undertook to create an Educational Scorecard to track performance towards the goal of achieving our section’s shared educational vision

Using the work of Ogrinc et al. [10] as a guide, the task force brainstormed a broad set of potential metrics (48 total) balanced across the four domains of the Educational Value Compass and from each of the six perspectives from which the value of education should be assessed. Experts then participated in a modified Delphi technique [11] to build consensus of opinion on the most valuable metrics to measure successful attainment of our efforts. In our first round of the Delphi process we presented our experts with the 48 potential metrics and asked them to rate each as “Not useful”, “Potentially useful but wording unclear” or “Clear and useful”. We also asked the experts to provide us with any suggestions for metrics that they did not see on our list. Based on expert ratings we discarded seven potential metrics rated as “Not Useful”. We created operational definitions for 41 remaining metrics as well as for five additional metrics suggested by our experts in Round One. For Round Two, we asked our experts to rate each of these 46 metrics on a Likert Scale ranging from 1 to 5 with anchoring comments where 1 was “Not Useful”, 3 was “Moderately Useful” and 5 was “Very Useful”. Expert responses from this round provided the basis for rank ordering of the metrics. We selected the top two ranked questions from each perspective. In examining the results we noted three highly ranked items in the Clinician-Teacher and Learner perspectives and no highly ranked items in the Community perspective. The task force elected to include the higher ranked responses from the Teacher and Learner perspectives while omitting the Community perspective. This resulted in a final list of 12 metrics balanced across five of the six perspectives articulated by Ogrinc et al. [10]. See Fig. 1 for a high-level summary of the development process.

### Scorecard implementation

Of the 12 metrics identified, five had existing data sources, while seven required new data collection. Systems were developed to collect this data, including the dissemination of an annual survey to all faculty. The datasets generated and analyzed during our work are available from the corresponding author on reasonable request. We found no pre-established benchmarks for any of our metrics. So, we developed performance targets for each metric by analyzing historical trends and aligning with agreed upon goals and tracked the sectional performance within the metric over time.

## Results

### Scorecard evaluation

Our educational scorecard was designed in 2014. After one year of data collection (Academic Year 2014–2015), the scorecard was presented to all sectional members (*n* = 30). Sectional participation in the process was high with roughly 90% of sectional members completing the survey. The scorecard was felt to represent a broad and transparent view of our educational impact and enabled our section and section chief to suggest strategic ways to better meet our educational mission based on our data.

### Sectional performance

Data for eight of the 12 metrics was available for the first year of analysis; our section failed to meet performance targets for seven of the eight metrics, and partially met target for the eighth. After changes at the individual and the sectional level (described below), our sectional performance improved in seven of the eight metrics, meeting performance targets in four of the eight metrics for which there was two years of data, and eight of the twelve metrics overall resulting in a more balanced educational impact (Table 1).

**Table 1:**
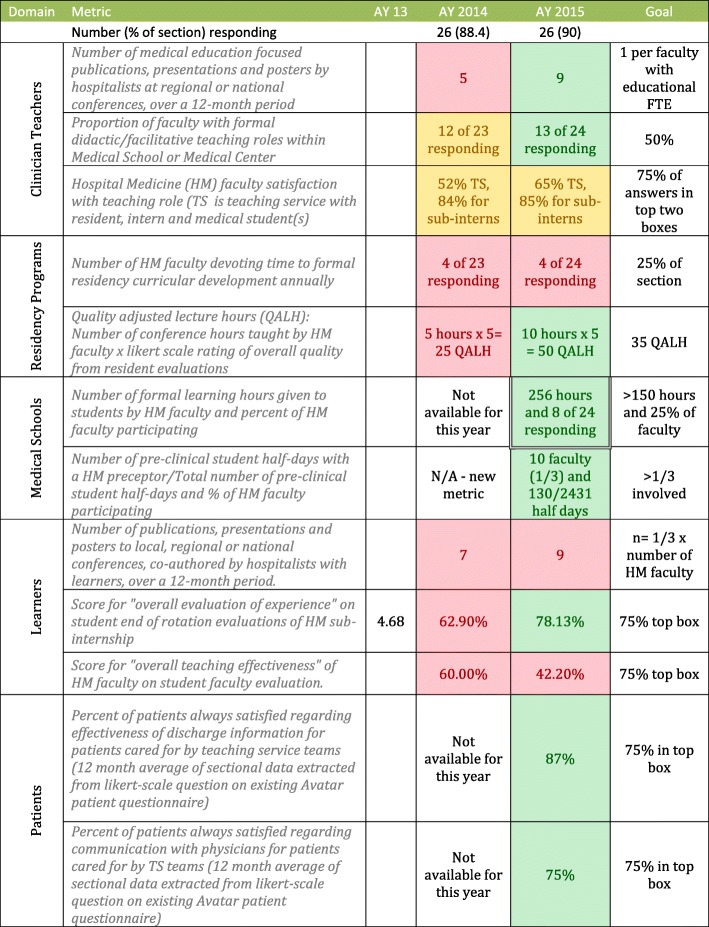
Educational Scorecard: Multi-year display of our section’s performance in 12 different metrics relative to performance goals allowing us to track performance towards the goal of achieving our section’s shared educational vision

Color coding used to denote progress in specific areas: green = on target, red = off target, yellow = borderline performance

### Sectional changes

As part of the scorecard review and survey process, faculty members provided suggestions on how to improve the educational experience and opportunities available to the section. Suggestions that were deemed desirable and feasible by the majority of the group were implemented as a result of this process. We identified seven sectional-level changes occurring as a result of the scorecard, which led faculty and our section to better meet our mission and vision. These included a change in teaching service structure to increase continuity with learners and an addition of medical student sub-interns to a hospitalist team that had previously been staffed only by physicians. Our section chief began scheduling faculty to teaching and non-teaching services based on faculty ability and interest in education –matching those interested in education with learners and those most interested in service on the non-teaching teams. Organization of the introduction to clinical medicine course was re-structured to allow increased participation of hospitalists. Additional impacts of the scorecard include an ongoing improvement in the number of metrics in which we achieved target performance, an improved ability to attract new hospitalists interested in actively engaging with our educational mission and vision, an improved orientation for new hospitalists emphasizing educational expectations, and improved sectional cohesion and interest in the educational mission as demonstrated by dedicated time at regular section meetings to focus on the section’s educational mission, with improved attendance at this meeting.

### Individual changes

The section’s endorsement of the scorecard and its associated metrics provided members with a more tangible list of educational goals. Our first survey focused on gathering data to determine whether we were meeting the performance targets for each metric. For our second annual survey, we added questions surrounding the surveying process, and individual involvement/engagement in educational opportunities. Junior faculty responded with multiple requests to be engaged with specific educational opportunities, such as “I would love an opportunity to give lectures.” And “Primary interest is undergraduate medical education – interested in collaborating on curriculum ideas/pilots.” Members of the scorecard development team met with each faculty member indicating an interest in further educational engagement, and developed plans to assist them in achieving their individual goals. Specific impacts on individuals include successfully funding protected educational time for three additional faculty members of the section, and presence of faculty members on multiple influential medical school committees such as the Medical Education Committee (controlling the curriculum of the medical school), the Committee on Student Performance and Conduct, and the Faculty Council.

## Discussion

After a year of development and two years of utilization, our scorecard has become an essential part of sectional improvement and has led to multiple positive impacts on the section and its members (described above), bringing us closer to achieving our educational mission and vision. Our group of performance-driven faculty, used to metrics tracking performance in revenue generation, clinical productivity, and meaningful use, responded positively and enthusiastically to metrics that track performance in education. We anticipate the scorecard to continue to be utilized, but as we achieve our performance targets year after year, certain metrics may be replaced by others the section wishes to target. As it is currently configured, our scorecard is comprised of lagging measures, which often measure outcomes. Ideally, our scorecard would also mature to include more leading metrics, such as participation of members in educational faculty development, which can predict trends and drive performance [12].

Our project was conducted in a single section at a single institution, and the metrics we generated may not be appropriate or generalizable to other institutions. Our aim in disseminating our work was not to focus on the specific metrics included in our scorecard, but instead to describe the process by which we developed the scorecard. Each section or division focusing on education has its own culture and objectives and we hope other groups focusing on educational improvement would employ the method we used successfully to generate different metrics based on the mission and vision they aim to achieve. In addition, given that our metrics lend themselves to yearly data collection and changes made on an annual basis, evaluating changes piloted as a result of this project requires an extended time beyond the scope of this paper. We plan to provide follow-up about the longitudinal feasibility and results of our educational scorecard at a later date.

In our experience, the process by which we developed this tool provided the most value to our section. When presented at a national meeting, there was much interest in directly adopting the specific metrics within our tool in order to benchmark between organizations. While this is tempting, we would not expect that simply adopting our scorecard would lead to success at other institutions, but we believe that our process can be easily adopted by other sections, divisions or institutions desiring to advance their educational mission and vision. The task force found the process overall to be manageable. The process of identifying and surveying experts required a modest investment of time; however, the remainder of the work moved relatively quickly with a minimum of time invested beyond the normal work expectations within an academic section. We completed this work without direct funding by the department.

## Conclusion

We have found our Educational Scorecard to be an efficient and useful way to gather and display data about how the section is performing in achieving its educational goals. Our educational scorecard directly motivated sectional leadership and sectional members, individually and as a group, to prioritize our educational mission and vision at a time of multiple competing interests, and has continued to motivate and instigate positive change over time. An educational scorecard is a feasible way for academic groups to communicate educational goals and current group performance, engage faculty, and provide objective information with which to base strategic decisions affecting the educational mission.

## Abbreviations

FTE: Full-time equivalent
HM: Hospital Medicine
QALH: Quality-adjusted lecture hours
RVU: Relative Value Unit
TS: Teaching Service
